# Fourier Transform Mid-Infrared Spectroscopy (FT-MIR) as a Method of Identifying Contaminants in Sugar Beet Production Process—Case Studies

**DOI:** 10.3390/molecules28145559

**Published:** 2023-07-20

**Authors:** Radosław Michał Gruska, Alina Kunicka-Styczyńska, Andrzej Jaśkiewicz, Andrzej Baryga, Stanisław Brzeziński, Beata Świącik

**Affiliations:** Department of Sugar Industry and Food Safety Management, Faculty of Biotechnology and Food Science, Lodz University of Technology, ul. Wólczańska 171/173, 90-530 Lodz, Poland; alina.kunicka@p.lodz.pl (A.K.-S.); andrzej.jaskiewicz@p.lodz.pl (A.J.); andrzej.baryga@p.lodz.pl (A.B.); stanislaw.brzezinski@p.lodz.pl (S.B.); beata.swiacik@p.lodz.pl (B.Ś.)

**Keywords:** infrared spectroscopy, sugar quality, white sugar contaminants, food safety, sugar industry

## Abstract

Food safety has received considerable attention in recent years. Methods for rapid identification of a variety contaminants in both the final product and the manufacturing process are constantly developing. This study used Fourier Transform Mid-Infrared Spectroscopy (FT-MIR) spectroscopy to identify various contaminants endangering white sugar production. It was demonstrated that inorganic compounds (calcium carbonate—CaCO_3_), plastic contaminants (polypropylene), and oily contaminants (compressor sealing and lubrication lubricant) can be identified with a high degree of precision. FT-MIR spectroscopy was proved to be a useful technique for detecting sugar contaminants rapidly and precisely even without the application of a sophisticated spectra analysis. Commercial databases of reference spectra usage significantly simplify and facilitate the application of this method.

## 1. Introduction

In both the sugar beet and sugarcane industries, there are no standardized techniques for detecting varieties of inorganic compounds, plastic, and oily contaminants. The analytical techniques adapted for the sugar industry are developed and validated by the International Commission for Uniform Methods of Sugar Analysis (ICUMSA) and primarily include quantitative analyses of already-known impurities. For example, spectrophotometric techniques are used to determine the amount of selected metals, e.g., zinc and cadmium [1], or assess the content of starch and dextran [2,3]. Reducing sugars are analyzed quantitatively by titration [4]. If extraneous contaminants occur, there is no officially accepted method of their identification, but varied techniques are used for quantitative analysis. Water-insoluble substances are quantitatively determined by filtration or gravimetric methods [5] and also as conductometric ash [6,7].

The FT-MIR technique has not been used as an official analytical technique in the sugar industry and such an application is still under research. Officially, only a related technique that relies on infrared light is used, namely NIR polarimetry, which can be used for polarimetric sucrose content estimation in raw sugar [8], Pol and Brix determination in sugarcane and other confectionery products [9], or to determine the content of sucrose, dry substance, and fiber in sugarcane [10]. The expanding technological line for sugar beet processing raises the danger of introducing impurities of various origins and makes it difficult to identify and select the appropriate analytical technique. In addition, most of the more versatile techniques, such as Atomic Absorption Spectrometry (AAS) or chromatography, are time-consuming and often require complex sample preparation. The Fourier Transform Mid-Infrared Spectroscopy (FT-MIR) technique does not require complex treatments in sample preparation, and the analysis itself, spectra comparison, and results assessment take a short time, which is a huge advantage in technological conditions, when quick intervention often avoids deterioration of the product.

Fourier transform infrared spectroscopy (FT-IR) uses the interference between two IR beams to yield a signal (interferogram), which is a function of the change in wavelength between the two beams. The signal usually generated by a Michelson interferometer is transmuted by Fourier transform algorithms [11]. The Fourier transformation converts the interferogram’s intensity versus time to an IR spectrum of intensity versus frequency [12]. Formerly, the resolution of most FT-IR instruments was comparable to dispersive IR instruments [13], and they were not often utilized also because of the high cost. However, once the bottleneck of computational power was overcome, the increased resolution outweighed the cost, and FT-IR became increasingly used [14]. The infrared region of the electromagnetic spectrum extends from visible light to microwave radiation. In the analytical technique, infrared radiation is divided into three ranges: near infrared (NIR, ν = 10.000–4.000 cm^−1^), middle infrared (MIR, ν = 4.000–200 cm^−1^), and far infrared (FIR, ν = 200–10 cm^−1^). Different compounds can be analyzed both quantitatively and qualitatively by FT-IR spectroscopy due to their characteristic absorption/emission in the IR spectral region [15]. Mid-infrared spectroscopy (MIR) is based on the absorbance of IR radiation of dipole bonds within functional groups of molecules. The absorbance of IR radiation excites the electrons in the bonds to a higher state of vibration, causing bending or stretching motions of the molecular bonds [12,14,16]. A molecule will absorb IR radiation only if the absorption of the particular wavelength causes a change in the at-rest or excited vibrational states [14,17]. Most chemical compounds express a dipole moment and can be identified by their characteristic infrared absorption [14,17]. The MIR spectrum is divided into four major regions: stretching (4000–2500 cm^−1^), triple bonds (2500–2000 cm^−1^), double bonds (2000–1500 cm^−1^), and fingerprint (1500–400 cm^−1^). In the fingerprint region, unique absorbance patterns of each sample are presented, allowing the distinction of similar substances [18]. For organic compounds, characteristic stretching vibrations usually occur between 4000 and 1500 cm^−1^, in the so-called MIR region [12,14]. However, below 1500 cm^−1^, vibrations, generally bending, result in a characteristic fingerprint of the molecule or a large part of the molecule and can therefore give structural information useful for identification [11,14]. The absorbance intensity is directly related to the concentration of a pure compound or functional group when analyzing mixtures [12,14], making IR spectroscopy suitable for quantitative purposes. FT-MIR spectroscopy allows the analysis of solid, liquid, or gaseous samples using different sampling techniques (transmission or reflectance) that improve the resolution of the obtained spectra.

Since the FT-IR technique is fast and allows precise identification of the type of material being tested, attempts are made to use it for the rapid identification of impurities and contaminants in food, industry, or the environment. Microplastics in food, water, or the environment can be found using FT-IR [19,20,21,22,23,24]. Fourier mid-infrared (FT-MIR) and near-infrared (FT-NIR) spectroscopy may also be used in food quality control [25,26,27,28,29,30,31]. Arzu Yazici et al. [32] established a method for rapid nondestructive detection of pesticide residues in strawberries based on the near-infrared spectroscopy. Visible-NIR spectroscopy combined with chemometric methods was used to quantify chlorpyrifos and carbendazim residues in cabbage [33]. A method based on the radial basis function (RBF) neural network combined with FT-NIR spectral data was applied to assess the talc content in wheat flour [34]. Vis-NIR reflectance spectroscopy based on physical property analysis was employed to estimate the concentration of azodicarbonyl in flour [35]. The usefulness of infrared spectroscopy for detection of toxic substances in food was also documented [36]. Infrared spectroscopy may serve a reliable applicable method for a nondestructive detection of various types of undesirable food ingredients. 

The use of infrared spectroscopy as an analytical technique has also been investigated for the sugar industry requirements [37]. The FT-IR spectroscopy was used to detect the sucrose content or physical properties and moisture in beets [38,39,40,41]. Moreover deterioration effects in sugar beet during storage were successfully monitored by FT-NIR [42]. Tannin, lignin, and wax content were determined in sugarcane using the FT-NIR method [43]. Raw and purified (filtered, saturated) thin and thick sugar juices and diluted massecuites were investigated using the transmittance method. Apart from these, Brix value (dry matter) and sugar content, color, as well as microbiological contamination of juices were measured with acceptable accuracy [44,45,46,47,48].

As the preceding literature data demonstrate, infrared spectroscopy is an adaptable technique and can be used to determine a fairly broad spectrum of chemical compounds. The aim of the study was to apply the FT-MIR technique to identify contaminants introduced during the process of white sugar production from sugar beets. Three cases of sugar contamination in white sugar production have been illustrated.

## 2. Results and Discussion

### 2.1. Case 1—Determination of the Presence of Calcium Carbonate in White Sugar

**Figure 1:**
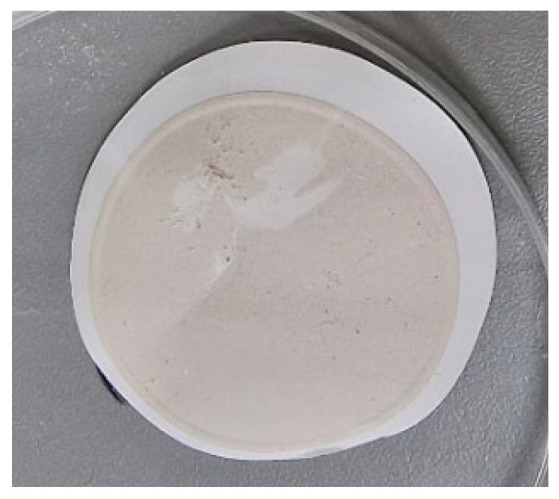
Contaminant precipitate (Sample A) after filtering of white sugar water solution.

**Figure 2:**
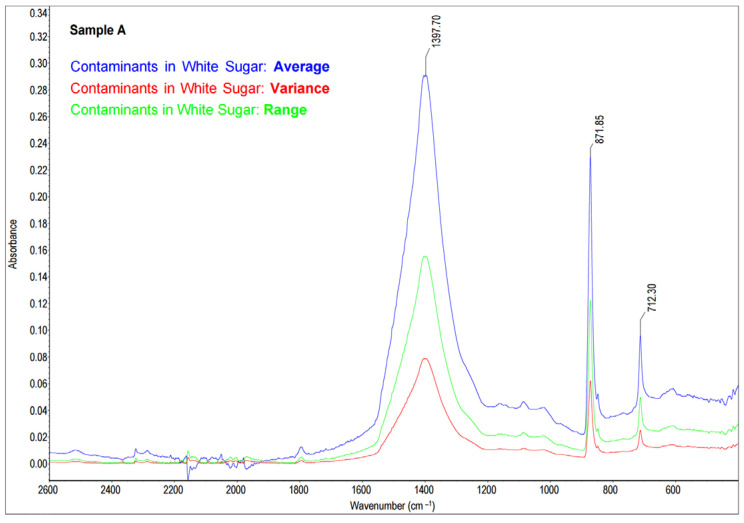
The statistical analysis of the FT-MIR spectra of contaminants in white sugar (number of samples = 3, total number of scans = 96; for each data point: average—the arithmetic mean of the Y values; variance—the standard deviation of the Y values; range—the margin of Y values).

External, water-insoluble contamination (Sample A) was detected in white sugar. After the dissolution of sugar in water and filtration, a creamy-white precipitate was left on the filter surface (Figure 1). Since the nature and the origin of the contamination was difficult to identify on the basis of macroscopic features, the mid-infrared (MIR) spectroscopy technique was used. The collected precipitate was mixed with KBr, and a pellet was prepared (as described in Section 3.3). The obtained spectra were statistically analyzed in accordance with Section 3.4, and the results are presented in Figure 2. 

**Figure 3:**
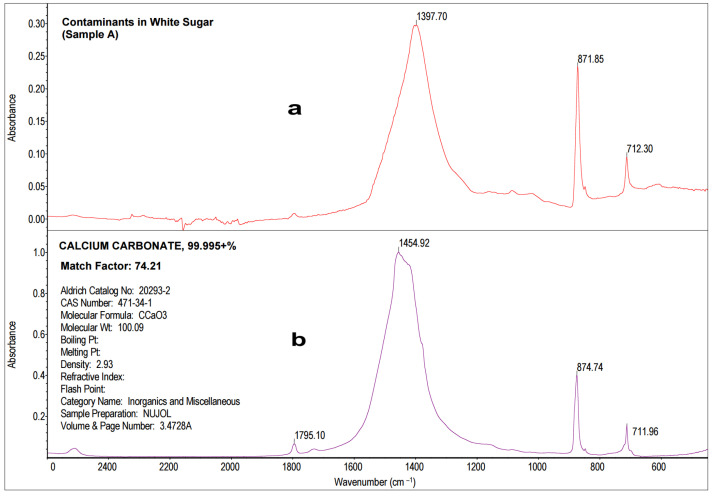
The MIR spectrum of the white sugar water solution precipitate (Sample A) (**a**) compared to spectra from a database of HR Aldrich Organometallic, Inorganics and Miscellaneous, Aldrich Catalog No. 20293-2 (**b**).

The resulting spectrum was compared to the spectra in the standard library (Section 3.3 and Section 3.4) and presented in Figure 3. A comparative analysis of Sample A revealed that the tested precipitate was identified as calcium carbonate (CaCO_3_) with a 74% Match Factor according to the spectrum from the database of HR Aldrich Organometallic, Inorganics and Miscellaneous (Aldrich Catalog No. 20293-2). The spectra similarities were observed in the wavelength number range from 1800 cm^−1^ to 600 cm^−1^. In (a) and (b) spectra, there are three distinct absorbance peaks: at a wavenumber of about 1400–1450 cm^−1^, at a wavenumber of about 870–875 cm^−1^, and at a wavenumber of about 710–713 cm^−1^ (Figure 3). According to the literature [49,50], the observed vibrations (peaks) testify the presence of a carbonate group CO_3_^2−^ (asymmetric C-O stretching at wavenumber of approximately 1400–1450 cm^−1^), out-of-plane vibration (the wavenumber of approximately 870–875 cm^−1^), and in-plane vibration (at a wavenumber of approximately 710–713 cm^−1^) associated with calcite (CaCO_3_ polymorph). The last peak may be representative for dolomite or calcite, rocks and minerals from the group of carbonates, composed of calcium carbonate (CaCO_3_) predominantly. The white sugar prevailing contaminant was identified as calcium carbonate.

Based on the result of the analysis, the source of sugar contamination in the production process was recognized. In sugar technology during a juice purifying process, calcium compounds are used. The calcium carbonate (limestone) is burning to produce burned lime in a lime kiln located in the sugar factory. The capacity of the lime kiln often exceeds 100 m^3^ (usually 150–300 m^3^), so large amounts of limestone were processed. As open containers are used to transport limestone to the top of the kiln, large quantities of fine dust may be produced and spread with the wind in the premises of the production plant. The rapid identification of contaminants has enabled specific preventive action to be taken. The use of special partitions protected white sugar from calcium carbonate dust entering the warehouses and sugar contamination.

### 2.2. Case 2—Identification of Lubricating or Sealing Oil Entering the Beet Cossettes

**Figure 4:**
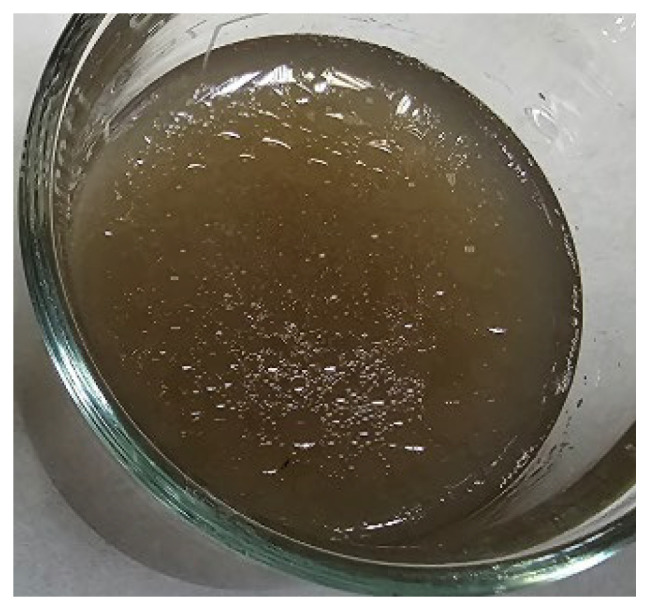
The oily contaminants of cossettes mixed with water (Sample B).

Prior to the sucrose extraction, sugar beet roots are cut into cossettes to intensify the diffusion process. Slicing machines currently used in the sugar industry have a slicing capacity from 3000 to about 8000 tons per day (t/d) [51]. Due to the need to ensure a sugar factory processing continuity of production of 12,000 tons of beet roots per day, there should be two in-use machines and one as a reserve. In the presented case, the traces of oily substance was noted after the cossettes made contact with water. Figure 4 illustrates the oily substance after mixing the cossettes with water.

**Figure 5:**
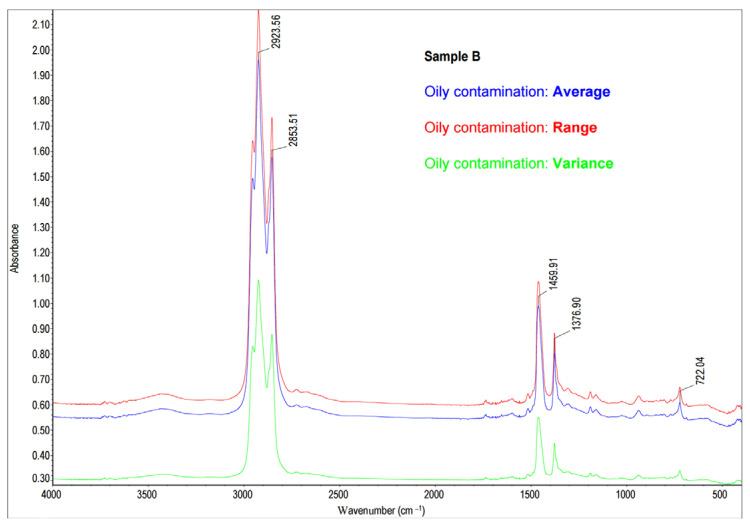
The statistical analysis of the FT-MIR spectra of oily contamination in the cossette (number of samples = 3, total number of scans = 96; for each data point: average—the arithmetic mean of the Y values; variance—the standard deviation of the Y values; range—the margin of Y values).

**Figure 6:**
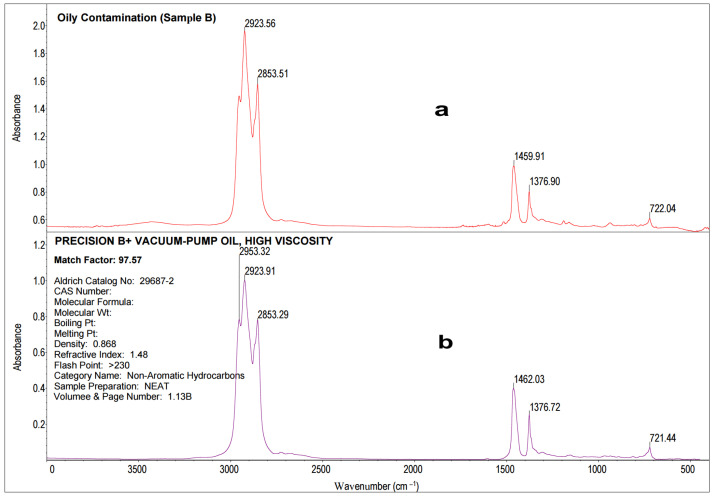
The MIR spectrum of the oily cossette contaminant (Sample B) (**a**) compared to spectra from a database HR Aldrich Hydrocarbons, Non-Aromatic Hydrocarbons (**b**).

In order to locate the leakage of oily substances, a quick identification of contaminant components was inevitable. These impurities may not only adversely affect the subsequent processing steps but may also impair the quality of white sugar. Since the literature data show IR spectroscopy as one of the quickest methods for oil identification [52,53,54,55,56], this technique was also applied in this particular case. A sample of oil contamination (Sample B) was collected from the water’s surface and placed on a measuring window of IR sample cards (Real Crystal^®^) as described in Section 3.3. Figure 5 presents the statistical analysis, and the average spectrum was compared to the spectra in the standard library, HR Aldrich Hydrocarbons. The oily contaminant of the cossette (Sample B) and the MIR spectra of the oil described as Precision B+ Vacuum-Pump Oil High Viscosity were of high similarity (97.6% Match Factor) (Figure 6). 

The reference spectrum originated from a library of Non-Aromatic Hydrocarbons, Aldrich (Catalog No: 29687-2). Precision B+ Vacuum-Pump Oil High Viscosity is mainly used to seal and lubricate air compressors. The spectrum of Sample B enabled us to infer the chemical structure of this oil. In the range of 2900–2800 cm^−1^, bands from CH bond stretching vibrations of saturated n-alkyl groups can be observed with absorption maxima at wavenumbers 2924 cm^−1^ and 2835 cm^−1^, which correspond to the base lubricating oil hydrocarbons [57,58]. The deformation vibrations of the CH bonds exhibiting a band with a peak at 1462 cm^−1^ and 1377 cm^−1^ belonged to the CH_2_ and CH_3_ groups and indicate the base lubricating oil hydrocarbons [57,58]. The frequency of 721 cm^−1^ on the spectrum corresponded to the vibrations of the CH bonds of the long carbon chains, typical of poly-alpha-olefins [57,58]. Zzeyani et al. [58] found that changes in the composition of the lubricating oil during its operation may be indicated by the evolution of these bands, testifying the lubricant degradation.

The slicing machine usually undergoes periodical cleaning. In the sugar factory, the cleaning is mainly realized with compressed air. The application of oil compressors instead of oil-free compressors may bring the risk of oily contaminations. Owing to the flawed compressor, some oil leaked out with the compressed air, contaminating the slicing machine. As a result, the oil penetrated the cossettes during slicing.

### 2.3. Case 3—Polypropylene Identification in Sugar Dust

**Figure 7:**
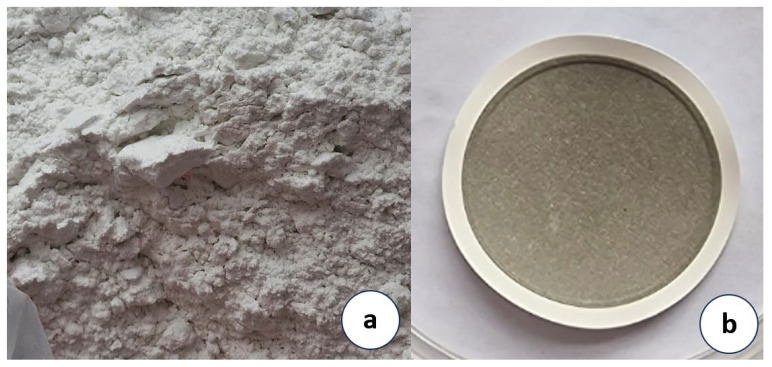
White sugar dust (**a**) and the contaminant residues after filtering of sugar dust water solution (Sample C) (**b**).

Sugar factories producing crystal sugar from both sugar beets and sugarcane generate significant quantities of sugar dust (Figure 7a). The dust is a highly valuable raw material utilized to enhance the factory’s economic performance and productivity. Routinely, it is dissolved in water up to a certain sucrose concentration and returned to the production process.

In Case 3, a significant quantity of insoluble contaminants of unknown origin appears in the sugar dust water solution. The contaminants formed a layer on the filter (Figure 7b). As the precipitate during blending with KBr (to form pellets) was of plastic character, it was decided to measure the reflectance using an ATR attachment (as described in Section 3.3). For this purpose, 0.1 g of sediment (Sample C) was put on the measuring window, and the reflectance was measured.

**Figure 8:**
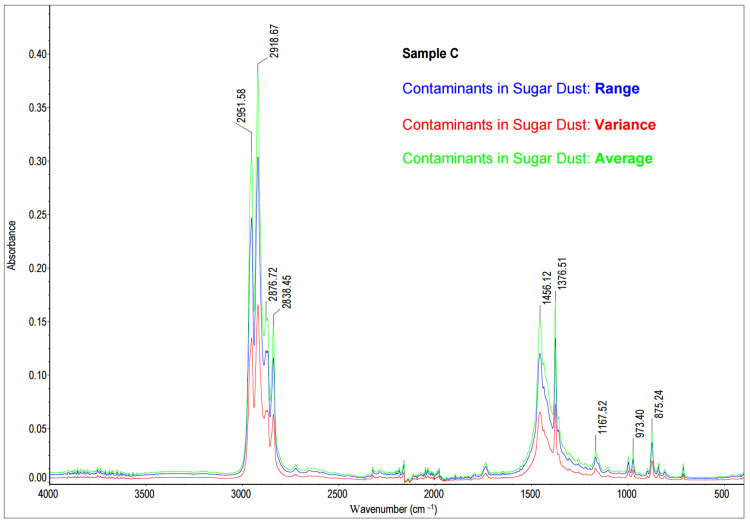
The statistical analysis of the FT-MIR spectra of contaminants in sugar dust (number of samples = 3, total number of scans = 96; for each data point: average—the arithmetic mean of the Y values; variance—the standard deviation of the Y values; range—the margin of Y values).

The results of the statistical analysis are presented in Figure 8.

**Figure 9:**
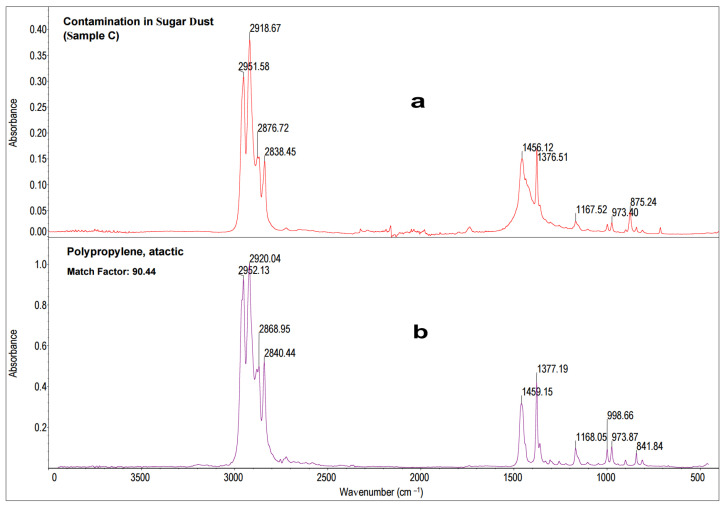
The MIR spectrum of the sugar dust contaminant (Sample C) (**a**) compared to spectra from a database of reference spectra, HR Nicolet Sampler Library (**b**).

The obtained spectrum of the precipitate (Figure 9a) was compared to a database of standard spectra, HR Nicolet Sampler Library (Figure 9b). A comparative analysis of Sample C, sugar dust contaminant, revealed the tested precipitate consisted most likely of polypropylene (90, 44% Match Factor). The reference spectrum for polypropylene is derived from a database of reference spectra named HR Nicolet Sampler Library. Analysis of the characteristic absorption bands confirmed that the test sample is composed of polypropylene [59,60,61,62,63]. In the ranges of 2951–2952 cm^−1^ and 2868–2877 cm^−1^, there were absorption bands from asymmetric and symmetric stretches CH_4_ [49,50,51,52,53]. Absorption bands in the ranges of 2918–2920 cm^−1^ and 2868–2877 cm^−1^ originated from asymmetric and symmetric stretches CH_2_ [59,60,61,62,63]. The absorption bands 1456–11459 cm^−1^ indicating CH_2_ deformation or bend were found [59,60,61,62,63]. Moreover, wavenumbers 1377–1377 cm^−1^ absorption bands from CH_3_ umbrella mode were also noted. Absorption bands typical of isotactic polypropylene bands include 1167–1168 cm^−1^, 999 cm^−1^, 973 cm^−1^, and 842 cm^−1^ [59,60,61,62,63].

Identifying the contaminants as polypropylene simultaneously allowed for the determination of the contamination’s source. Polypropylene is utilized as the compound of the sugar conveyor belts not only for its mechanical but also electrostatic properties. Polypropylene as a dielectric polymer under external mechanical force can generate energy by the coupled effect of contact electrification and electrostatic induction between two materials (triboelectric effect) [64]. Electrical discharges produced by the conveyor belt and transported sugar may bring the risk of an explosion [65]. Sugar crystals transported by conveyors acquire a negative charge, so the belts are made of materials that also accrue negative charge in order to prevent electrical discharges [64]. The identification of polypropylene as the sugar powder contaminant made it possible to identify the locations of damaged parts of the sugar conveyor belts.

## 3. Materials and Methods

### 3.1. Samples

Case 1: Crystal white sugar was the final product of a sugar beet factory. The sugar was collected from a bucket conveyor, between the sugar dryer and the packaging equipment. A sample of 5 kg of sugar was taken and thoroughly mixed prior to further analysis. Sample A—contaminants of white crystal sugar.

Case 2: A cossette was collected from slicing machines cutting the roots of sugar beet. A total of 10 kg of cossettes were collected and mixed thoroughly prior to the analysis. Sample B—oily substances from beet cossette.

Case 3: Sugar dust was produced during sugar transportation. An amount of 5 kg of sugar dust was collected from conveyor belts and bucket conveyors. Sample C—contaminants of white sugar dust.

### 3.2. MIR Apparatus

All analyses were carried out by means of the Thermo Scientific^TM^ Nicolet^TM^ iS50 FT-IR Spectrometer (Thermo Fisher Scientific Inc., Waltham, MA, USA). MIR spectra were recorded and analyzed using OMNIC 9.3.30 software (Thermo Electron Corporation, Waltham, MA, USA). FT-MIR analyses were carried out with a DTGS KBr detector, IR light source, and KBr beamsplitter (integral parts of iS50 FT-IR Spectrometer). The range of scans was 4000–400 cm^−1^ with a resolution of 4.00 cm^−1^. Each spectrum was derived from 32 scans of the sample. The different measurement modes were used: transmittance measurements for Cases 1 and 2 and attenuated total reflectance (ATR) for Case 3. 

### 3.3. Samples Preparation

Contaminant samples (Samples A and C) were prepared in the following manner: 500 g of sugar or sugar dust was dissolved in 1500 g of distilled water at 50 °C. A total of 1000 cm^3^ of the solution was collected and filtered (MF-Millipore^®^ Membrane Filter, 8 µm pore size, 47 mm, Merck, Darmstadt, Germany), with filtration under a pressure of 100 Pa. The filter was then rinsed with 1000 cm^3^ of deionized water and dried. The residues that remained on the surface of filters were used for analysis. Measurements of transmittance/absorbance were used for Sample A examination. For this purpose, 1 mg of Sample A and 199 mg KBr (analytical grade) were mixed and pressed into a pellet at 17 kN. A pure KBr pellet was a reference to examine the contribution of KBr contamination to the spectrum in the range of OH stretching vibrations. Measurements of reflectance were applied for Sample C analysis. The built-in mid-IR-capable diamond ATR module was used: 0.1 g of Sample C was applied to the measuring window, and the reflectance was measured.

For oily contaminants (Sample B), the procedure was as follows: 1000 g of the cossettes were placed in a beaker with 3000 cm^3^ of distilled water. After stirring the contents, the beaker was placed in an ultrasonic cleaner (Sonic-10, POLSONIC Palczyński Sp. J.) for 15 min. The floating contaminants were carefully collected. An amount of 0.1 cm^3^ of the sample was taken for testing, and the transmittance/absorbance was measured by means of IR sample cards (Real Crystal^®^, International Crystal Laboratories, New York, NY, USA) with KBr glass (9.5 mm aperture).

### 3.4. Data Processing

The identification of contaminants was carried out using OMNIC 9.3.30 software (Thermo Electron Corporation, Waltham, MA, USA). In each case, three samples of contamination were collected, and each sample was scanned 32 times. In the next step, OMNIC 9.3.30 statistical analysis was performed on the collected spectra. The results of the statistical calculation were plotted in a spectral window using a vertical axis that indicates the average, variance, and range. The average is the arithmetic mean of the Y values for each data point. The variance is defined in OMNIC software as the standard deviation of the Y values for each data point. The range is the Y_max_ and Y_min_ margin value for each data point. The obtained average spectra were compared with the reference spectra database provided by the device’s manufacturer (Thermo Electron Corporation, Waltham, MA, USA). The bases were provided by Sigma-Aldrich Co. LLC, Burlington, MA, USA (database: HR Aldrich) or by Thermo Fisher Scientific Inc., Waltham, MA, USA (database: HR Nicolet Sampler Library). The degree of similarity between the analyzed spectra and the reference spectrum was determined by the OMNIC software automatic expert mode. Identification was considered satisfactory if the correlation coefficient (Match Factor) between the spectrum of the test sample and the standard spectrum exceeded 0.70 [66,67].

## 4. Conclusions

Infrared spectroscopy was found to be a useful technique for contaminant detection in sugar beet production. This study used FT-MIR spectroscopy to identify three types of sugar contaminants and helps in the location of their source in the production process. The presented research confirmed the high suitability and efficacy of FT-MIR spectroscopy in identifying mineral (calcium carbonate), synthetic (polypropylene), and oily (compressor lubrication and sealing oil) contaminants with a few simple steps. Although the case studies described concerned the sugar industry, this flexible technique may be utilized in other food industries. The only requirement for its effective and accurate application is the sufficiently large database of reference spectra enabling the reliable identification of the tested sample. It is important to acknowledge limitations of this technique. One of the primary limitations of FT-MIR spectroscopy is its sensitivity to water. Since water has strong absorption in the mid-infrared region, samples with high water content can pose challenges in obtaining accurate spectra [1]. Another limitation is the complexity of the spectra obtained from FT-MIR spectroscopy. If the sample has a complex composition, the spectra often contain overlapping absorption bands, which can make it difficult to identify specific compounds or contaminants without the use of complex data analysis techniques [68]. The assessment of the suitability of the FT-MIR technique for the determination of extraneous contaminants will be further analyzed focusing on the exact development of quantitative methods, along with a thorough assessment of the sensitivity, detection limit, and scope of application for selected impurities. Further research will be also focused on broadening the range of analyzed matrices.

## Data Availability

Data available on request due to restrictions, e.g., privacy or ethical. The data presented in this study are available on request from the corresponding author. The data are not publicly available because they are part of the authors’ own research.

## Sample Availability

Samples of the compounds are available from the authors.
